# Recent advances in the degradation of polytetrafluoroethylene (PTFE) at low temperature (≤100 °C)†

**DOI:** 10.1039/d6sc03192g

**Published:** 2026-06-11

**Authors:** Matthew E. Lowe, Matthew N. Hopkinson, Dominik J. Kubicki, Erli Lu, Roly J. Armstrong

**Affiliations:** a School of Natural and Environmental Sciences, Newcastle University Newcastle Upon Tyne NE1 7RU UK roly.armstrong@newcastle.ac.uk; b School of Chemistry, University of Birmingham Edgbaston Birmingham B15 2TT UK d.j.kubicki@bham.ac.uk e.lu@bham.ac.uk; c Birmingham Centre of Mechanochemistry and Mechanical Processing, University of Birmingham B15 2TT UK

## Abstract

Polytetrafluoroethylene (PTFE) is a polymeric material that displays remarkable thermal and chemical stability. These properties have allowed for the widespread application of PTFE in both everyday and specialist environments but come at a price when the time comes for its disposal. PTFE is highly persistent in landfill, pyrolysis generates low molecular weight ‘forever chemicals’ and forcing conditions are typically required for its mineralization. Recently, these challenges have inspired intense research to discover new methods for the low-temperature degradation of PTFE. In several cases, the fluoride that is liberated can be upcycled through incorporation into fluorinated fine chemicals. This perspective explores the cutting-edge methods for the degradation of PTFE at low temperature and, in some cases, subsequent chemical upcycling.

## Abstract 2

This perspective provides an overview of recent methods (since 2023) enabling the degradation of PTFE at low temperature (≤100 °C) forming useful fluoride salts, which in some cases can be upcycled into valuable fluorinated fine chemicals.

## Graphical abstract

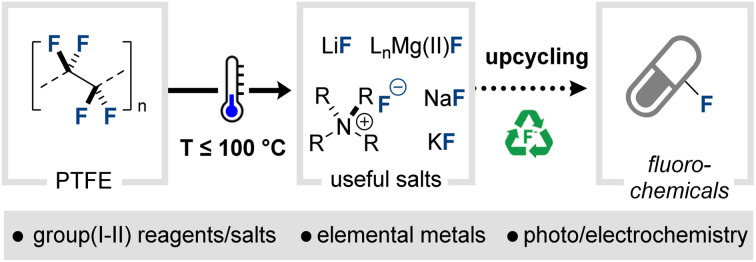

## 1 Introduction

**Scheme 1:**
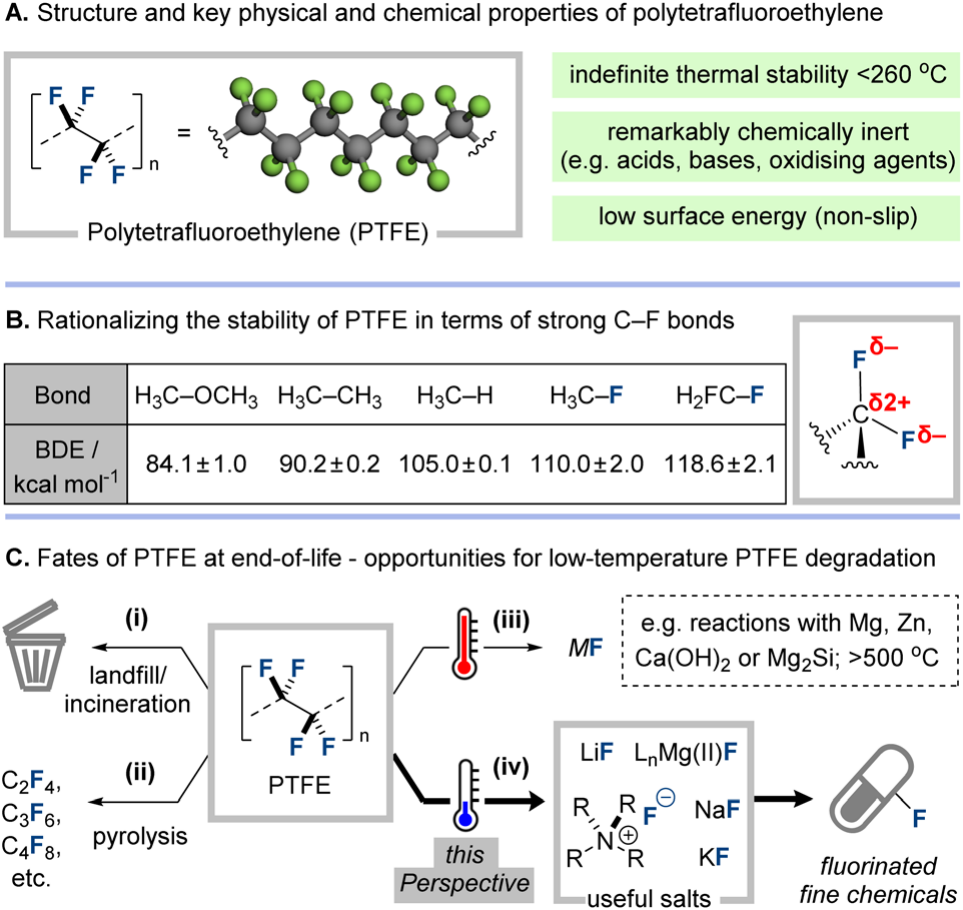
Structure and key properties of PTFE, the chemical origins of its stability, and associated challenges and opportunities for its disposal. M = metal.

Polytetrafluoroethylene (PTFE), more commonly known as Teflon™, is a versatile fluoropolymer first discovered in 1938 at the chemical company DuPont (Scheme 1A).^1^ Today PTFE is produced on a very large scale (global production of 309 000 tons in 2021),^2^ and is used in a wide variety of applications including non-stick coatings on cookware and textile products, as well as more specialized uses in healthcare, communications, aerospace, electronics, and construction. The wide-ranging utility of PTFE is owed to its remarkable properties: it is thermally stable at temperatures up to 260 °C (and for short periods of time up to 400 °C),^3^ and is also exceptionally chemically robust, resisting degradation from most Brønsted acids and bases, nucleophiles, oxidising agents and solvents. The low surface energy of PTFE also results in some of the lowest friction coefficients of any solid material – an order of magnitude lower than that of most other polymers – making it ideal for a variety of non-stick applications.^4^

These chemical and physical properties of PTFE arise from its unique perfluorinated structure. The highly polarized carbon–fluorine bond is the strongest single bond in organic chemistry, with bond dissociation energies typically in excess of 100 kcal mol^−1^, reflecting a substantial ionic contribution to bonding (Scheme 1B).^5^ Moreover, in molecules such as PTFE where a single carbon is substituted with multiple fluorine atoms, the strength of each individual C–F bond becomes even greater, as seen in the enhanced C–F bond dissociation energy in difluoromethane (118.6 kcal mol^−1^) over its monofluorinated analogue (110.0 kcal mol^−1^).^5*a*^ This has been rationalized on the basis that the additional fluorine atom renders the carbon atom even more electron deficient (*i.e.* formally C^*δ*2+^), thereby strengthening the electrostatic component to bonding with the partially negatively charged fluorine atoms.^5*b*^ It is these very strong C–F bonds in PTFE, in conjunction with the ability of the partially negatively charged fluorine atoms to form a protective sheath around the carbon backbone, which are the critical factors driving its high chemical and thermal stability.

These characteristics are crucial for the applications of PTFE, but they pose a significant challenge once the material reaches the end of its lifecycle.^6^ The majority of waste PTFE is incinerated or sent to landfill (Scheme 1C, i), where it persists for long periods of time, undergoing minimal biodegradation.^7^ Alternatively, PTFE can be pyrolyzed at high temperatures, but this can lead to the generation of shorter chain polyfluoroalkyl substances (PFAS) *e.g.* C_2_F_4_, C_3_F_6_, C_4_F_8_*etc* (Scheme 1C, ii).^3,8^ There is growing concern and regulatory legislation surrounding PFAS, which have been shown to be detrimental to human health in some cases and are highly persistent in the environment, earning them the moniker ‘forever chemicals’.^9^ Accordingly, there has been a great deal of interest in finding alternative methods to degrade PTFE, particularly so-called mineralization approaches capable of cleaving the strong C–F bonds to produce relatively environmentally benign inorganic fluoride salts. A variety of such processes have been developed, but they typically require extremely high temperatures, forcing conditions and/or specialized techniques, for example the use of supercritical water or CO_2_ (Scheme 1C, iii).^10^

**Scheme 2:**
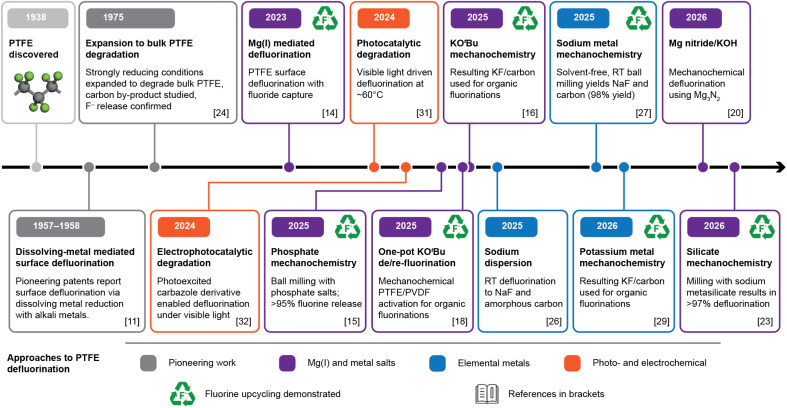
Selected chronology and milestones in low temperature PTFE degradation and fluorine upcycling.

Overcoming these challenges to devise methods for PTFE degradation at low temperature (defined for the purposes of this perspective as ≤100 °C) has attracted substantial attention (Scheme 1C, iv). Pioneering work in the late 1950s aiming at surface modification of PTFE to improve its susceptibility to adhesion identified that treatment with alkali metals in liquid ammonia at room temperature results in degradation of the PTFE surface, releasing water soluble fluoride (Scheme 2).^11^ Following this discovery, a series of elegant reports emerged extending this chemistry to surface or bulk PTFE degradation with other dissolving metal reductions as well as group-1 metals (molten, vaporized or as dispersions in organic solvent) or their amalgams. These early methods, which are summarized in Section 3, release metal fluoride salts along with formation of carbon-rich residues. The predominant focus of most of this early work was upon characterizing and studying the materials properties of the carbonaceous products formed rather than the released fluoride component. Recent developments have witnessed rapid growth in this area, with the emergence of numerous powerful approaches capable of efficiently releasing fluoride from PTFE at temperatures ≤100 °C, which is the focus of this perspective. These methods have been shown to liberate a variety of useful fluoride salts, which in several instances have been applied to perform fluorination reactions of various small molecule building blocks, thereby allowing the fluorine atoms from PTFE to be upcycled into valuable fluorochemicals.^12,13^ These recent approaches are summarized chronologically in Scheme 2. They include the application of group (I) or (II) metal salts or coordination complexes (purple boxes), elemental metals (blue boxes), as well as electro- and photochemical methods (orange boxes). The article is broken down according to these three different classes of reagent and surveys both the methodology employed for PTFE degradation, as well as fluoride upcycling where relevant. The focus is upon methods that have been developed within the past three years (since 2023), although key prior art is summarized where relevant. The article is centred on PTFE, but selected examples involving other fluoropolymers and small-molecule PFAS are included where they provide mechanistic context, illustrate closely related degradation chemistry, or demonstrate fluoride upcycling strategies relevant to PTFE.

## 2 Degradation with low-valent magnesium species and alkali metal salts

**Scheme 3:**
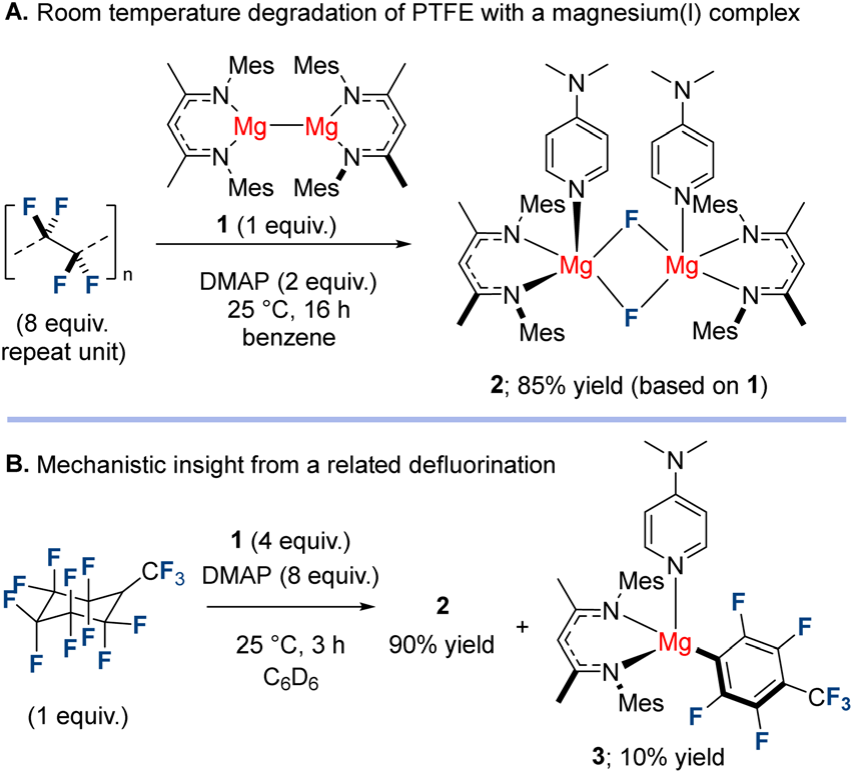
Reactions of a low-valent magnesium(i) complex with PTFE or perfluoromethylcyclohexane reported by Crimmin and co-workers.^14^ DMAP = 4-dimethylaminopyridine; Mes = 2,4,6-Me_3_-C_6_H_2_.

A seminal contribution in this area was made by Crimmin and co-workers in 2023, who reported the reaction between a dimeric, low-valent magnesium(i) complex 1 with a large excess of PTFE in the presence of 4-dimethylaminopyridine (DMAP), in benzene at room temperature, which led to the formation of a dimeric magnesium(ii) fluoride complex 2 in 85% yield (Scheme 3A).^14^ This yield is calculated on the basis of the stoichiometrically limiting complex 1 and corresponds to release of ∼6% of the fluorine content from the PTFE. The resulting, partially degraded PTFE was analysed by XPS, SEM, PXRD, IR and solid-state NMR, from which the inference was made that degradation occurred predominantly at the surface of the polymer. Of note, the IR spectrum of the partially degraded PTFE appeared to show new C=C stretching and bending modes, implying the formation of alkene groups on the surface of the PTFE. This assignment was supported by hydroboration of the partially degraded PTFE with BH_3_·THF in THF for 72 hours, which resulted in the disappearance of the vibrations assigned to C=C bonds in the IR spectrum. The group went on to perform a reaction between a stoichiometrically limiting quantity of perfluoromethyl(cyclohexane) with magnesium(i) complex 1 (4 equiv.) and DMAP (8 equiv.). This resulted in generation of complex 2 in 90% yield, but also the appearance of complex 3 in 10% yield (Scheme 3B). The generation of this perfluoroaromatic complex 3 further supports the possibility of defluorination to form C=C bonds. To provide additional evidence for this, the group used DFT calculations to investigate the mechanism for the defluorination of C_2_F_6_ (a simplified model for PTFE or perfluoromethylcyclohexane). These calculations support the feasibility of an overall 1,2-defluorination process occurring *via* stepwise addition of a dimeric, monoligated magnesium complex to a C–F bond followed by elimination of fluoride. The magnesium fluoride complex 2 was shown to be soluble in organic solvents and could be used to fluorinate both Me_3_SiCl and BF_3_, representing an early demonstration of the potential for fluorine upcycling.

**Scheme 4:**
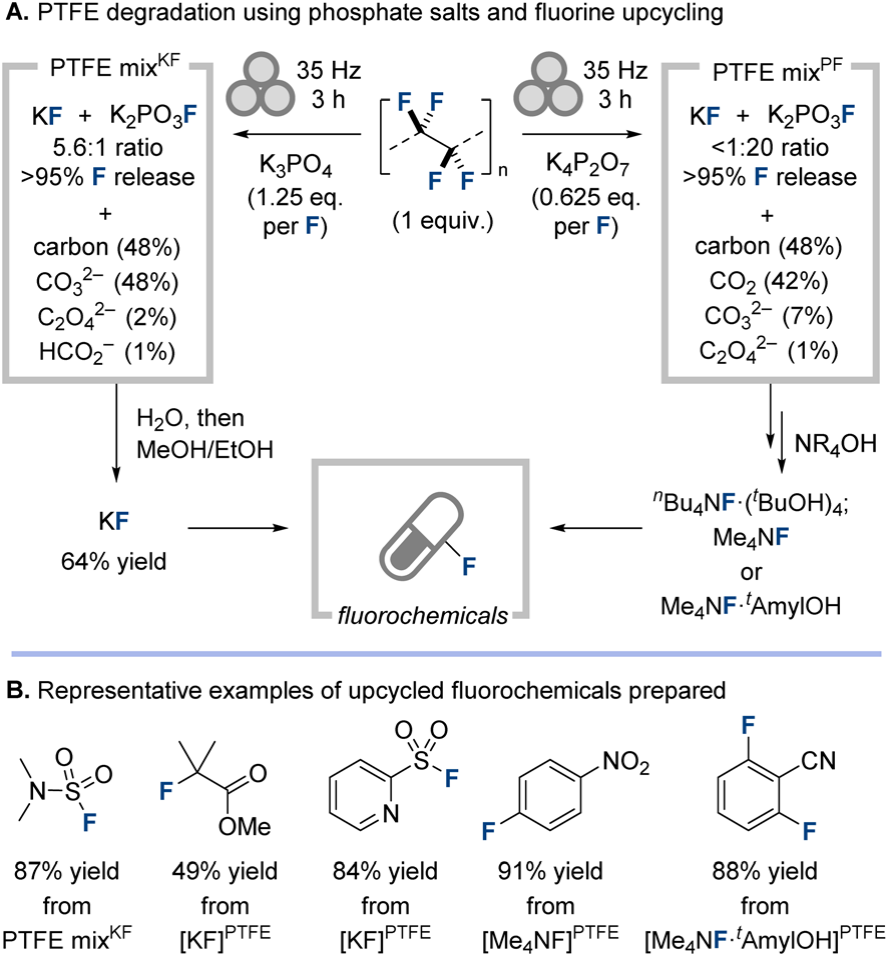
The degradation of PTFE with phosphate salts reported by Gouverneur and co-workers.^15^ Only the fluorine- and carbon-containing components of “PTFE mixtures” are shown for simplicity.

In March 2025, Gouverneur, Paton and co-workers reported a pioneering mechanochemical procedure for the degradation of PTFE with phosphate salts (Scheme 4A).^15^ Two protocols were developed in which PTFE was ball-milled at room temperature with either K_3_PO_4_ or K_4_P_2_O_7_, resulting in the release of >95% of the fluorine content of the polymer in both cases. The resulting solid residues, denoted “PTFE mix^KF^” and “PTFE mix^PF^” respectively, contained either potassium fluoride or potassium fluorophosphate as the principal fluorinated species, together with non-fluorinated byproducts such as carbon, carbonate, CO_2_, bicarbonate, pyruvate, phosphate and pyrophosphate. This procedure took only three hours and worked well for PTFE in the forms of powder, tape, and a seal. The ability of the approach to tolerate non-powdered PTFE is particularly attractive, and may be assisted by the solvent free, high-concentration, milling process which can constantly expose fresh PTFE to the phosphate salt and facilitate a greater rate of reaction. These PTFE mixes could be used to synthesise well-established fluorinating agents such as KF or tetraalkyl ammonium fluoride salts, which were isolated and then applied in a variety of downstream substitution reactions, thereby achieving indirect upcycling of fluoride into sulfonyl fluorides, alkyl fluorides, and (hetero)aryl fluorides (Scheme 4B). It was also found that the crude PTFE mix^KF^ could be applied directly as a fluorinating agent for S–F bond construction, for example the synthesis of *N*,*N*-dimethylsulfamoyl fluoride in 87% yield. Notably, the phosphate mediated degradation was not limited to PTFE and was shown to be capable of efficiently degrading other fluoropolymers (*e.g.* polyvinylidene fluoride, ethylene tetrafluoroethylene, polyvinyl fluoride, fluorinated ethylene propylene) as well as various small molecule PFAS including perfluorooctanoic acid (PFOA) and perfluorooctanesulfonic acid (PFOS).

**Scheme 5:**
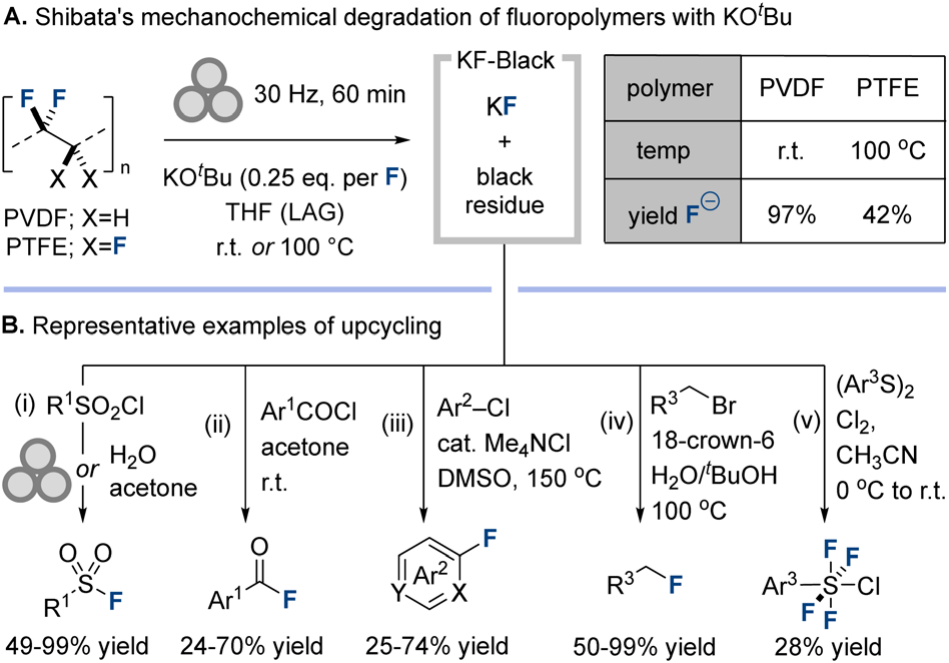
Mechanochemical degradation of PVDF and PTFE with KO^*t*^Bu reported by Shibata and co-workers^16^ followed by fluorine upcycling. THF = tetrahydrofuran; LAG = liquid assisted grinding.

Soon afterwards, in June 2025, Shibata and co-workers reported the mechanochemical degradation of fluoropolymers using potassium *tert*-butoxide along with a small quantity of THF to facilitate liquid assisted grinding (LAG, Scheme 5A).^16^ The main focus of this work was on the degradation of PVDF (polyvinylidene fluoride), which several previous studies had revealed to be susceptible to defluorination under basic conditions.^17^ Accordingly, ball-milling PVDF with a limiting quantity of KO^*t*^Bu (0.25 equiv. per fluorine atom) at room temperature delivered a black residue dubbed “KF-black”. Extraction with water followed by analysis *via* ion chromatography showed that a 97% yield of fluoride had been released with respect to KO^*t*^Bu. Interestingly, employing a slightly higher temperature of 100 °C, this protocol was also shown to be capable of degrading PTFE, generating KF in 42% yield. This is an intriguing observation given that unlike PVDF, PTFE lacks C–H bonds required to facilitate loss of fluoride through a conventional eliminative mechanism (*vide infra*). The KF-black generated upon defluorination of PVDF was shown to be a versatile reagent for nucleophilic fluorination (Scheme 5B). After drying under vacuum at 120 °C, it could be used without any further purification in an impressive range of reactions including: (i) nucleophilic fluorination of sulfonyl chlorides under either solution state or mechanochemical conditions; (ii) preparation of acyl fluorides from the corresponding acid chlorides; (iii) halex reactions of electron deficient heteroaryl chlorides at elevated temperature; (iv) substitution of primary alkyl bromides (tertiary alcohols could also be employed under acidic conditions); and (v) oxidative fluorination of an aromatic disulfide with chlorine gas in acetonitrile to give the corresponding SF_4_Cl derivative.

**Scheme 6:**
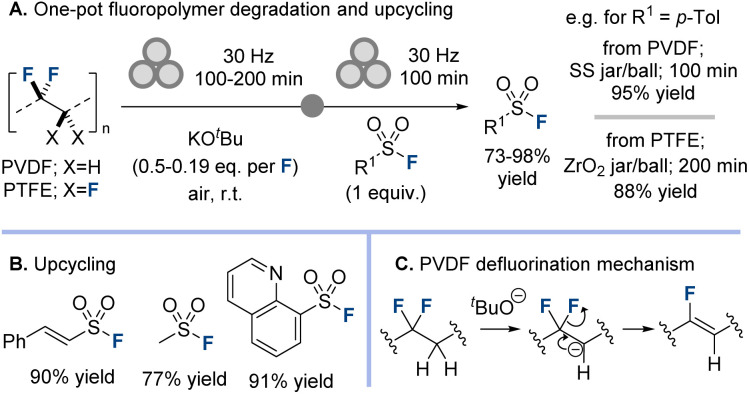
The one-pot mechanochemical defluorination–refluorination approach reported by Ackermann and co-workers.^18^ SS = stainless-steel.

Almost simultaneously, Ackermann and co-workers disclosed a very similar mechanochemical method for degrading fluoropolymers with KO^*t*^Bu (Scheme 6A).^18^ Again, the primary focus of this work was on the defluorination of PVDF, which was ball-milled in a 5 mL stainless-steel milling jar with a 7 mm stainless-steel ball along with 0.5 equiv. of KO^*t*^Bu per fluorine atom, no LAG, and a reaction time of 100 minutes. Depending on the stoichiometry and milling ball used, it was shown that 35–93% of the fluorine content of the PVDF was released as KF. The main focus of the work was on using an excess of the crude fluoride-containing residue in one-pot fluorination reactions. A wide scope of (hetero)aryl, vinyl and aliphatic sulfonyl fluorides could be obtained employing this approach in excellent yields (Scheme 6B). The authors also successfully expanded the mechanochemical degradation to PTFE, which, in line with Shibata's findings,^16^ proved to be a more challenging defluorination substrate. However, good reactivity could be achieved at room temperature by employing a larger milling jar (30 mL) and bigger ball (15 mm), both made from zirconia, in conjunction with a longer milling time of 200 minutes. This resulted in 28% release of fluoride, and the crude residue could be used to fluorinate tosyl chloride in an impressive 88% yield. The approach was also successful in upcycling other fluoropolymers and PFAS. Mechanistically, as shown in Scheme 6C, the authors proposed that PVDF is degraded *via* deprotonation by *tert*-butoxide to generate a carbanion, which can subsequently undergo β-fluoride elimination to form KF and dehydrofluorinated PVDF (dPVDF). This hypothesis is supported by the observation of a new C=C stretching peak at ∼1600 cm^−1^ in the FTIR of the crude residue after ball-milling. This mechanism is clearly not possible in PTFE, which lacks any sites for deprotonation. In this case it was postulated that the defluorination mechanism may proceed either *via* a single electron transfer pathway or through fluoride substitution.

Very recently, in February 2026, Crimmin and co-workers reported an elegant approach for solution-state degradation of hydrofluorocarbons with either KHMDS or KO^*t*^Bu employing THF as the solvent at 25 °C.^19^ Although not focussed upon PTFE and therefore strictly outside the scope of this perspective, it is interesting that there are several parallels with the work of Shibata and Ackermann described above.^16,18^ Hydrofluorocarbons (*e.g.* PVDF) were readily defluorinated at room temperature, with detailed computational and experimental studies supporting a similar, stepwise deprotonation–elimination mechanism to that outlined above in Scheme 6C. Moreover, fully fluorinated substrates such as PFOA, which are incapable of reacting *via* the same elimination mechanism, were nevertheless effectively degraded at higher temperature (150 °C).

**Scheme 7:**
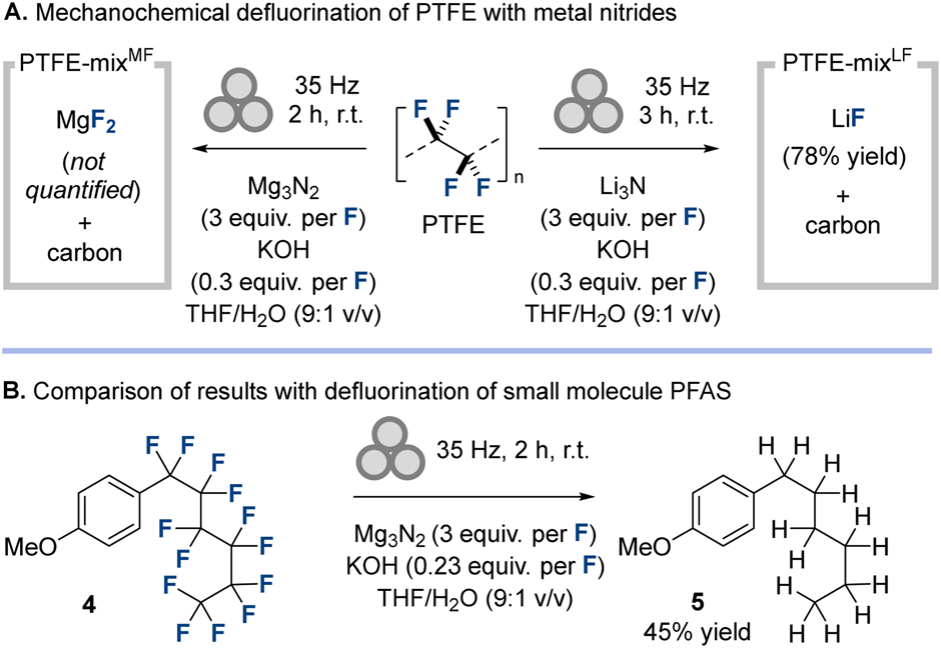
Nitride-mediated mechanochemical defluorination of PTFE and other fluorochemicals reported by Wang and co-workers.^20^

In March 2026, Wang and co-workers reported that PTFE can undergo mechanochemical defluorination in the presence of excess magnesium or lithium nitride along with a small quantity of KOH to afford residues which were referred to as “PTFE-mix^MF^” and “PTFE-mix^LF^” respectively (Scheme 7A).^20^ It was proposed that the Mg_3_N_2_ mediated degradation results in the formation of MgF_2_, although this was not quantified owing to its poor aqueous solubility. In the case of degradation with LiN_3_, quantitative ^19^F NMR analysis of the water-soluble fraction indicated that fluoride was released in 78% yield. The predominant focus of this work was not upon defluorination of PTFE, but rather of aryl fluorides and small-molecule PFAS, which was shown to result in protodefluorination (47 examples, 27–99% yield). For example, treatment of polyfluoroalkyl derivative 4 under very similar reaction conditions to those employed for the degradation of PTFE resulted in formation of the corresponding alkyl derivative 5 in 45% NMR yield (Scheme 7B). The different outcomes of defluorination observed for small molecule PFAS compared with PTFE is particularly noteworthy, with the former delivering fully saturated carbon products, but the latter generating partially reduced graphene (the authors comment that little or no polyethylene is generated in the process). More generally, this observation suggests that caution should be exercised when applying small molecule PFAS as simplified analogues to probe the mechanism of PTFE degradation protocols.

**Scheme 8:**
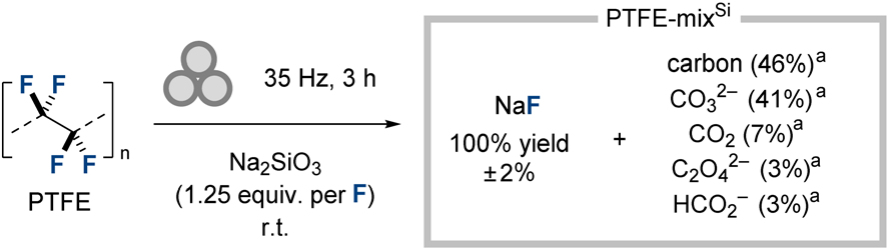
Gouverneur's silicate-promoted mechanochemical defluorination of PTFE. ^a^Refers to percentage of total carbon content.

Building upon important previous studies from Weber^21^ and Sperry^22^ describing SiO_2_-mediated defluorination of PFAS, in April 2026 Gouverneur and co-workers described a silicate-promoted mechanochemical defluorination of PTFE (Scheme 8).^23^ In this work, PTFE was ball-milled with a small excess of sodium metasilicate (1.25 equivalents per fluorine atom) at 35 Hz at room temperature for 3 hours delivering a solid residue referred to as “PTFE-mix^Si^”. Solid state ^19^F NMR spectroscopy of PTFE-mix^Si^ indicated a very high degree of mineralization had occurred, with 97% of NaF observed alongside trace amounts of residual PTFE (3%). The high efficiency of the process was confirmed by aqueous extraction followed by quantitative solution-state ^19^F NMR spectroscopy, which indicated a near quantitative yield of released fluoride (100 ± 2%). The fate of the carbon was more complex, with PTFE-mix^Si^ shown to comprise a water insoluble, black carbon-based component (46% of the total carbon content) along with water soluble components comprising predominantly carbonate along with trace amounts of C_2_O_4_^2−^, HCO_3_^−^ and gaseous carbon dioxide. The method was shown to be capable of efficiently degrading PTFE in various forms (*e.g.* seal or tape) as well as a broad range of other polymeric and non-polymeric PFAS.

## 3 Degradation by elemental metals

**Scheme 9:**
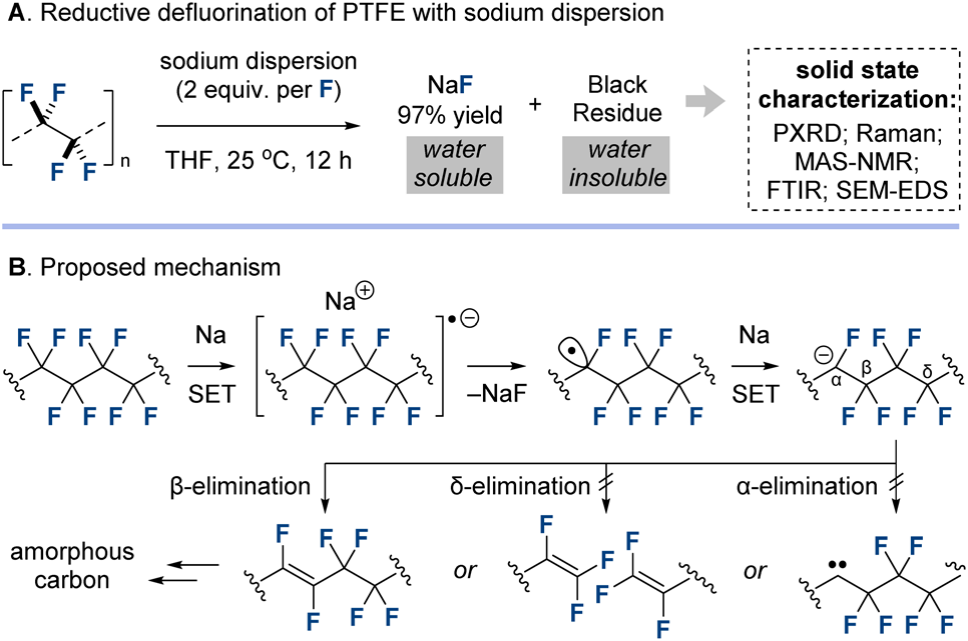
Defluorination of PTFE using sodium dispersion to give NaF and amorphous carbon, reported by Shibata and co-workers.^26^

For several decades, it has been known that group-1 or group-2 metals such as Li, Na, K, Cs, Mg and related single electron donors are some of the few chemicals capable of reacting with PTFE.^11,24^ Some of the earliest reported examples of low-temperature PTFE degradation involved application of group-1 metals in liquid ammonia to perform surface defluorination, thereby allowing the PTFE to be bonded more readily with adhesives.^11,24*a*^ Similar reaction conditions were subsequently expanded to perform defluorination reactions of bulk PTFE,^24*b*^ as well as degradations employing related reagents such as group-1 metal amalgams,^24*c*–*f*^ lithium or sodium naphthalenide,^24*g*–*i*^ benzoin dianion,^24*j*^ organolithiums,^24*k*^ along with a more recent example employing lithium and primary di- or triamines as a source of solvated electrons.^24*l*^ Metallic group-1 elements alone have also been shown to be capable of efficiently degrading PTFE, either as vapours deposited on the surface of the polymer,^11,24*m*^ or as dispersions in alkane solvents.^24*n*^ Thiebault and co-workers have also demonstrated that defluorination of PTFE with electrochemically generated magnesium is an effective method within the context of surface treatment.^24*o*^ Although fluoride release was confirmed in many cases, most of these early studies were predominantly focussed upon surface modification and/or characterizing the carbonaceous materials produced in these reactions and assessing their properties.^25^ The possibility of harnessing this reactivity to unlock practically useful synthetic defluorination protocols (and potential F-upcycling) was not realised until very recently. One of the biggest challenges is a practical one, namely how PTFE and an alkali metal, which are both insoluble solids in most common solvents (and reactive in the case of the latter) can be successfully brought together. A pioneering contribution in this area was made by the Shibata group, who in July 2025 reported a room-temperature degradation of PTFE employing sodium dispersion in THF. The process was highly efficient, employing only a small excess of sodium (2 equiv. per fluorine atom), to liberate 97% yield of fluoride (Scheme 9A).^26^ The water-insoluble black residue formed was analysed by PXRD, FTIR, Raman, ^19^F MAS-NMR, ^1^H MAS-NMR and SEM-EDS confirming it to be an amorphous, carbon rich material consistent with the C–F bonds in the PTFE having been reduced. These analyses also indicated the almost complete absence of PTFE in the amorphous carbon residue, again attesting to the high efficiency of defluorination. This procedure was applied to four other PFAS, including PFOA and TFA, requiring reaction times of 12 to 48 hours depending on the PFAS substrate and giving fluoride yields of 73–97%. The relatively long reaction times were attributed to the heterogeneous nature of the reaction, and as with such reactions, uniformity in stirring and scale were necessary for reproducible results. The group performed DFT calculations on short-chain model PFAS to determine a feasible mechanism for degradation (Scheme 9B). These studies support the reductive cleavage of a C–F bond *via* single electron transfer (SET) from sodium metal, which upon loss of NaF affords a carbon-centred radical. At this point, a second SET occurs to form a carbanion, which can eliminate a further equivalent of NaF. Several mechanisms for the elimination step were considered computationally, with the authors concluding that the most favourable option involves β-elimination to directly generate an unsaturated fluoropolymer, which was calculated to be significantly more favourable compared to alternative α- or δ-elimination processes. At this point the process can repeat to give near total defluorination.

**Scheme 10:**
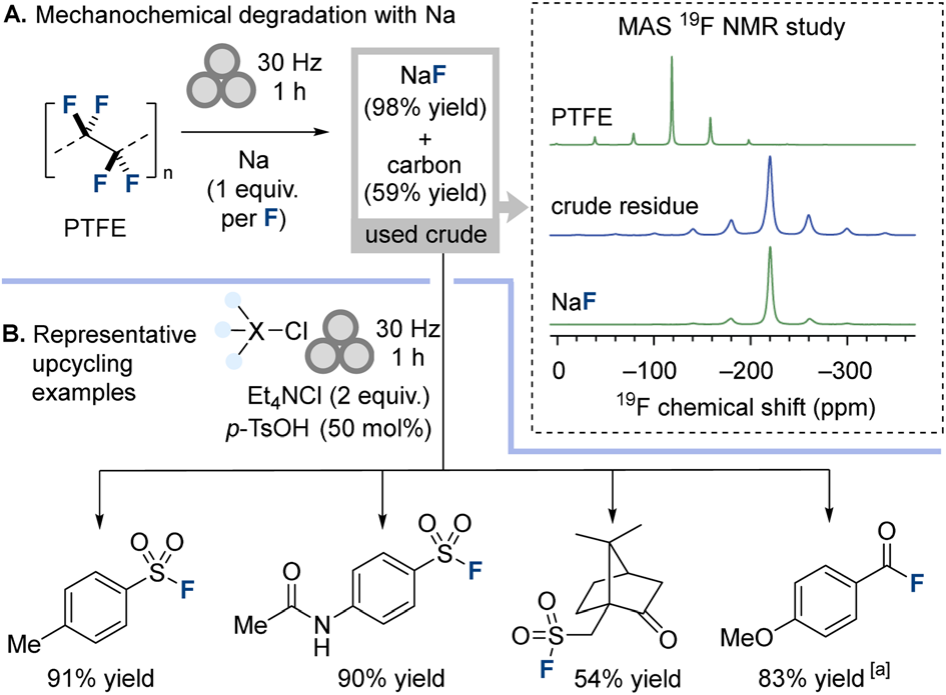
Mechanochemical degradation of PTFE with sodium metal followed by upcycling reported by Armstrong, Kubicki, Lu and co-workers.^27^^*a*^PPTS (1 equiv.) instead of *p*-TsOH.

In October 2025, the groups of Armstrong, Lu and Kubicki reported the reductive, mechanochemical degradation of PTFE with cheap, readily available chunks of sodium metal (Scheme 10A).^27^ The efficiency of this protocol is particularly noteworthy, with only 1.0 equivalent of sodium metal employed for each fluorine atom in the PTFE. After ball-milling at room temperature for one hour at 30 Hz, analysis of the crude residue by solid-state MAS ^19^F NMR spectroscopy revealed ≥99.9% conversion of the PTFE with new signals matching those of pristine sodium fluoride (Scheme 10A, inset). The NaF produced could be separated from the insoluble carbon-based byproduct by aqueous extraction and obtained in 98% NMR yield. Alternatively, the crude defluorination residue could be used directly in mechanochemical fluorination reactions with sulfonyl chlorides and acyl chlorides, delivering the corresponding fluorinated compounds in 33–95% yield (Scheme 10B). It was demonstrated that Brønsted acid and ammonium salt additives were essential to achieve efficient fluorination reactions, which is a consequence of the lower reactivity of NaF compared to KF. The authors also demonstrated that the procedure can degrade PTFE in a variety of different physical forms, including powder, tape or rod. This observation may reflect the capability of ball-milling to increase the surface area of the PTFE *in situ* allowing for full and rapid degradation.^28^

**Scheme 11:**
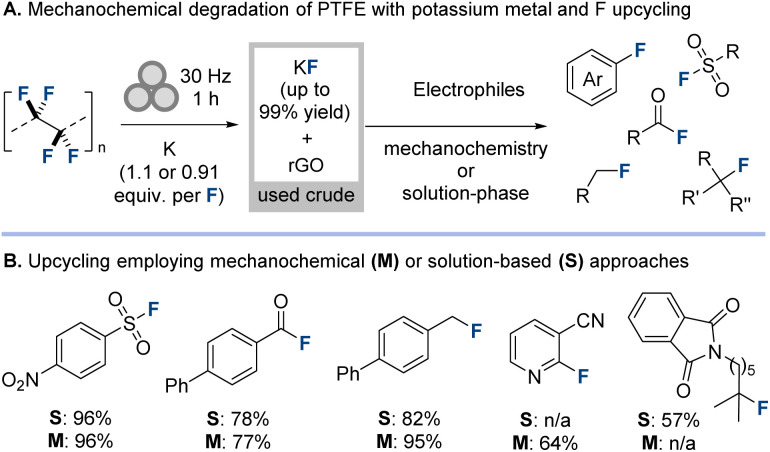
Shibata and co-workers'^29^ mechanochemical degradation of PTFE with potassium metal and selected examples of fluorine upcycling, in solution (S) or mechanochemically (M). rGO = reduced graphene oxide.

A further elegant mechanochemical method was reported in December 2025 by Shibata and co-workers, who demonstrated that potassium metal was also proficient in the degradation of PTFE.^29^ After ball-milling at room temperature for 1 hour, potassium fluoride was liberated in up to 99% yield alongside a black precipitate, which was identified as reduced graphene oxide (rGO) after washing with methanol and water (Scheme 11A). The crude KF_PTFE_–carbon mixture obtained from this mechanochemical reaction could be used without any purification in a variety of downstream fluorination reactions, including solution-state reactions with sulfonyl and acyl chlorides and substitution of primary alkyl bromides (a selection of examples are shown in Scheme 11B). Notably, all three of these transformations could also be performed mechanochemically, delivering the products in comparable yields. The synthesis of tertiary alkyl fluorides was successfully achieved under acidic conditions and, by adapting a protocol recently reported by Ito, Kubota and co-workers,^30^ it was also possible to apply the KF_PTFE_–carbon in mechanochemical nucleophilic aromatic substitution reactions of electron deficient aryl chlorides. The yields observed for fluorination reactions employing the KF/carbon mixture were shown to be comparable to those obtained with commercial KF. DFT calculations were performed for a short-chain analogue of PTFE to rationalize the mechanism of the K-mediated degradation. In line with the authors' previous approach employing sodium dispersion,^26^ these studies supported a mechanism involving two stepwise single electron transfers from potassium to PTFE, followed by β-elimination of the resulting carbanion. Interestingly, no intermediates were observed employing sub-stoichiometric quantities of potassium metal, leading the authors to propose that the initial C–F bond cleavage is the rate determining step, with subsequent defluorinations proceeding progressively faster.

## 4 Photo- and electrochemical degradation

**Scheme 12:**
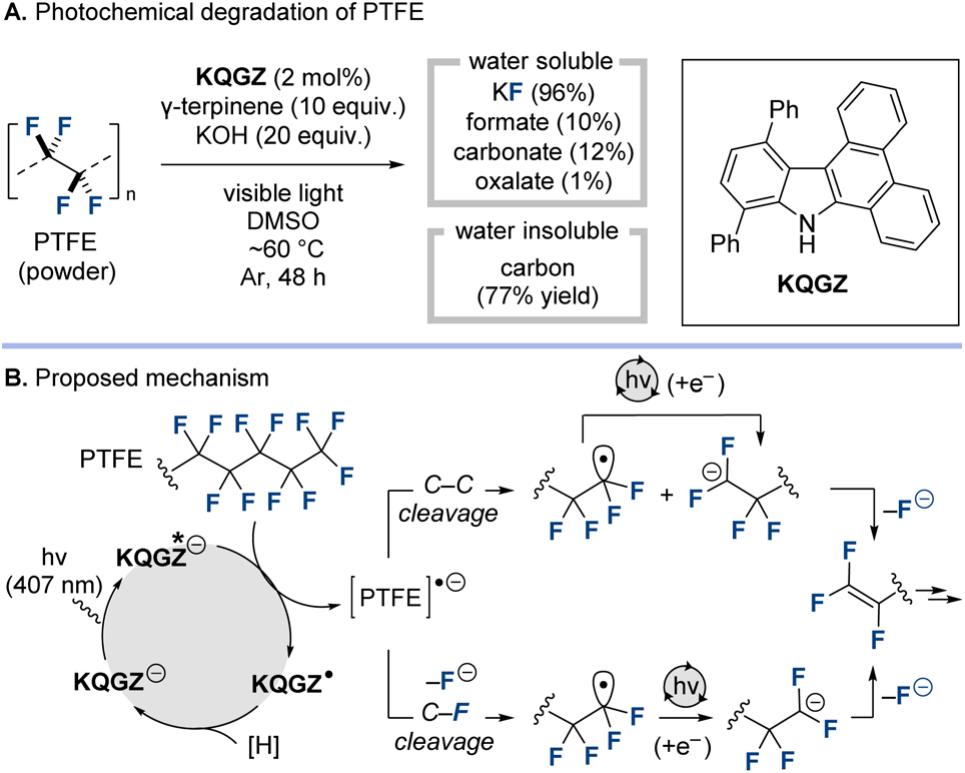
Kang and co-workers'^31^ photocatalytic degradation of PTFE.

In November 2024, Kang, Qu and co-workers reported a low-temperature, photocatalytic degradation of PTFE.^31^ The reactivity of 34 different photocatalysts with PTFE was investigated, with carbazole-derived photocatalyst KQGZ identified as optimal. Upon irradiation with visible light (*λ*_max_ = 407 nm) at ∼60 °C in DMSO, with KOH and a large excess of γ-terpinene (or cesium formate) as a terminal reductant, fluoride was liberated from PTFE in 96% yield (determined by solution state ^19^F NMR spectroscopy following aqueous extraction, Scheme 12A). The fate of the carbon was also studied with small amounts of water-soluble formate, carbonate and oxalate identified alongside formation of water-insoluble amorphous carbon in 77% yield. As might be expected given the heterogeneous nature of the reaction, several physical considerations were shown to be essential to achieve high efficiency, including careful positioning of the light source around the reaction vessel, a requirement for finely powdered PTFE (≤3 µm particle size), vigorous stirring (1000 rpm) and ultrasonication of the reaction mixture every 6–12 hours to prevent agglomeration. Based on a series of control experiments, the authors proposed the reaction mechanism shown in Scheme 12B. The process is initiated by photoexcitation of the deprotonated catalyst (KQGZ^−^) which can then transfer an electron to PTFE generating the corresponding radical anion [PTFE]˙^−^, along with KQGZ˙ which is recycled into its active form by the terminal reductant. Two potential defluorination mechanisms of [PTFE]˙^−^ were proposed: the first is directly analogous to that proposed by Shibata and co-workers,^26^ in which direct C–F bond cleavage occurs, releasing fluoride to generate a radical which can then be reduced to a carbanion *via* SET from the excited photocatalyst followed by a final β-fluoride elimination. Alternatively, a C–C cleavage event followed by SET and β-fluoride elimination could lead to an analogous perfluoroalkene intermediate. Repetition of these steps yields the final defluorination products.

**Scheme 13:**
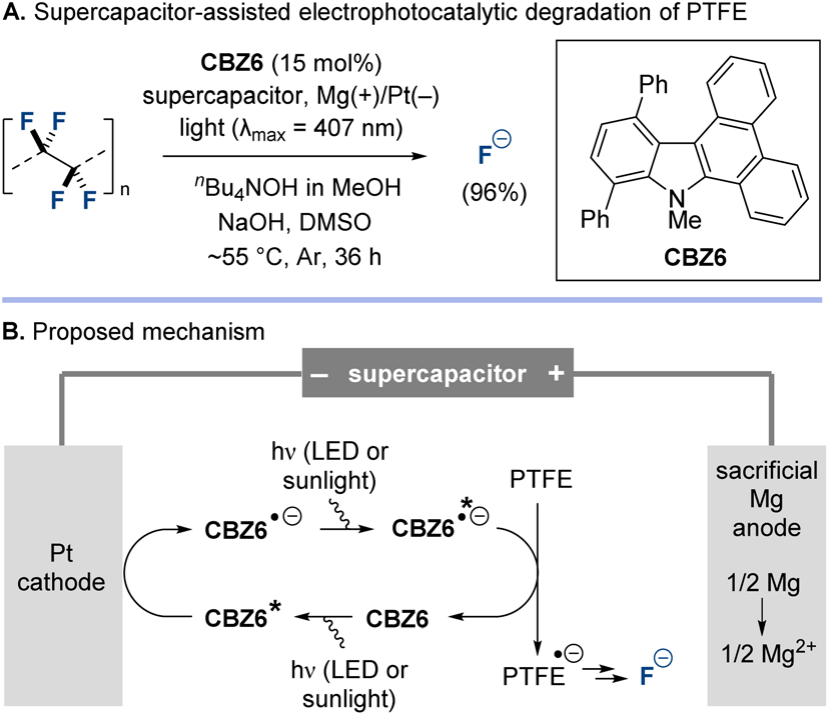
Electrophotocatalytic degradation of PTFE, facilitated by a supercapacitor and visible light, reported by Kang and co-workers.^32^

In March 2025, the Kang group reported a further study, aiming to address the requirement for a large excess of terminal reductant in their previous work.^32^ They demonstrated that this could be accomplished through electrophotocatalysis, in which visible-light photocatalysis and electrochemistry are performed simultaneously.^33^ A supercapacitor was employed as the electrical energy source with a platinum cathode and a sacrificial magnesium anode, which in conjunction with organic photocatalyst CBZ6 and irradiation with 54 W LEDs, was demonstrated to be capable of liberating fluoride from PTFE in up to 96% yield (Scheme 13A). A series of control experiments revealed that the photocatalyst, light, supercapacitor, electrolyte (tetrabutylammonium hydroxide) and sodium hydroxide are all essential for the reaction to occur. As with the previous method, the yield of the reaction was shown to be strongly dependent on the particle size of PTFE, with the best results obtained with particle sizes <1 µm. The portability of the supercapacitor enabled the setup to be taken outside and sunlight to be used as the light source which gave a 25% yield of liberated fluoride in 24 hours (3 × 8 hours). Mechanistically, the authors proposed that irradiation of CBZ6 with light leads to excited state species CBZ6* which can undergo single electron reduction at the cathode to form the corresponding radical anion CBZ6˙^−^ (Scheme 13B). At this point, a second photoexcitation generates [CBZ6˙^−^]* which was proposed to undergo single electron transfer with PTFE, returning the catalyst to its ground state and generating [PTFE]˙^−^. Repetition of this cycle to successively deliver further electrons, in conjunction with fluoride elimination eventually leads to near complete release of fluoride.

A growing body of other work, including recent elegant contributions from the groups of Lennox, Amanchukwu, Miyake, Paton, Damrauer and others has further highlighted the ability of photo- and electrochemical methods to perform reduction of other perfluorinated molecules.^34^ In conjunction with the work of Kang,^31,32^ these approaches are extremely attractive from a reactivity and sustainability standpoint. Their translation to larger scale will require careful consideration of solvent volume and recovery, additive and reductant/electrolyte loading, light penetration, electrode configuration, heat and mass transfer, PTFE particle-size requirements, and downstream product separation. Nevertheless, this growing body of work demonstrates that even extremely inert C–F bonds can be engaged through photo- and electrochemical strategies, pointing towards their significant potential for the remediation of perfluoroalkylated materials such as PTFE.

## 5 Summary, outlook and future opportunities

PTFE is a remarkably durable and resilient material which is crucial for our day-to-day lives. However, these same properties make the sustainable end-of-life management of PTFE a significant challenge. Increasing awareness of these issues means that ongoing use of PTFE (and other PFAS more generally) faces increasing scrutiny and regulation.^35^ In some cases, significant progress has been made in devising PFAS-free replacements for PTFE, for example in the development of ceramic, cast iron, and stainless-steel technologies for non-stick cookware.^36^ In parallel, it is essential to develop new ways to sustainably degrade PTFE waste, particularly given the huge amounts of PTFE already in circulation. The challenge here is the very high stability of PTFE, and even as recently as 2022, PTFE was widely regarded to be resistant to chemical transformation under mild conditions, with very few synthetically useful methods available for its low-temperature mineralization. The research summarized in this perspective highlights the dramatic progress that has been made in this area over the past three years, with numerous elegant methods now available to break down PTFE into useful fluoride salts at temperatures ≤100 °C. In general terms, almost all these strategies exploit the same chink in the armour of PTFE, namely its susceptibility to single electron reduction, which provides a means to cleave its strong C–F bonds, often forming fluoride salts with high lattice enthalpies in the process, thereby creating an enthalpic driving force. This includes a wide variety of creative chemistries, including the application of both bases and metals, as well as electro-, photo- and mechanochemical methods. In several instances, the fluoride released from the polymer has been applied, either directly or following purification, in downstream fluorination reactions, allowing it to be upcycled into valuable fluorochemicals.

In terms of future opportunities, it is instructive to consider the key attributes that determine the practical utility of a given method. In our opinion, these are:

(i) Achieving near complete defluorination of PTFE is critical to avoid generation of partially defluorinated intermediates. Such materials may themselves persist in the environment and continue to pose risks comparable to, or even exceeding, those of PTFE itself. This means that the fluorine mass balance of new processes must be carefully controlled and assessed, with a practical solution necessitating both highly efficient reactivity and operation with the fluoropolymer as the stoichiometrically limiting component.

(ii) The use of reagents which are safe, sustainable and commercially available at a relatively low cost.

(iii) The ability to effectively degrade PTFE of different grades and forms, for which mechanochemistry so far appears a particularly effective solution. This may be due in part to the ability of mechanochemistry to facilitate reactivity by breaking down larger PTFE particles into smaller ones and increasing surface area. However, it is important to recognise that the precise mechanism of mechanochemical activation is complex and not fully understood, with a number of other factors potentially playing important roles, including high effective concentration, efficient mixing, and potential to generate structural defects through mechanical stress.^37^

(iv) Going forward, developing methods capable of processing real-world waste streams in which PTFE is mixed with or coated onto other materials will also be an essential requirement. The ability to tolerate some exposure to water is a key consideration for remediating real-world PTFE waste streams. Perhaps most notably, the degradation of PTFE microplastics,^38^ an important class of persistent organic pollutants in water, would necessitate operation under aqueous conditions, a challenge that currently remains unmet by all methods discussed in this perspective.

(v) Generation of simple product mixtures facilitating upcycling of the fluoride produced, ideally without purification. Potassium fluoride-containing residues are particularly attractive for fluorine upcycling through synthetic organic chemistry – they have been employed in a wide range of nucleophilic fluorination chemistry, including the synthesis of sulfonyl fluorides, (hetero)aryl fluorides, acyl fluorides, SF_4_Cl derivatives, and primary or tertiary alkyl fluorides. Conversely, NaF and LiF are extremely important commodity chemicals, with applications in oral hygiene in the case of NaF, and as a precursor to LiPF_6_ for battery technology in the case of LiF.

(vi) The ability to efficiently and cost-effectively perform the chemistry at scale, which is essential given the multi-kiloton volumes at which PTFE is produced.

The numerous, powerful approaches for PTFE degradation surveyed in this perspective are collectively able to address many of these challenges. However, no one method is individually capable of fully satisfying them all. More work will be required before this can be achieved, and given the increasing concerns surrounding PTFE disposal, we anticipate that methodology development in this area will continue to intensify and grow. As more work is performed, there will also be opportunities to study the mechanism, kinetics and thermodynamics of PTFE degradation in more detail. The challenge of understanding these reactions lies in the insolubility of the polymer which limits *in situ* tracking of reactions by common analytical means. The polymeric nature of PTFE also makes explicit computational study challenging, as the long chain length would incur great computational costs, hence the studies of short-chain model systems used in the mechanistic explorations conducted so far. It is envisaged that continued exploration, particularly methods enabling *in situ* monitoring of reactions in the solid-state when applied to the degradation of PTFE, may allow deeper understanding of the underlying processes involved. Given the rapid advances of the past three years, we expect the coming years to be equally exciting.

## Author contributions

The content of this perspective was compiled following consultation between all authors. An initial draft was assembled by M. E. L. with subsequent refinements contributed jointly by all authors.

## Conflicts of interest

UK Patent application number 2603927.1, successfully filed with the UK Intellectual Property Office on 23 February 2026.

## Data Availability

No primary research results, software or code have been included, and no new data were generated or analysed as part of this Perspective.
